# *Bacillus cereus* Response to a Proanthocyanidin Trimer, a Transcriptional and Functional Analysis

**DOI:** 10.1007/s00284-016-1032-x

**Published:** 2016-04-09

**Authors:** Tomoko Tamura, Megumi Ozawa, Naoto Tanaka, Soichi Arai, Kiyoshi Mura

**Affiliations:** Department of Nutritional Science and Food Safety, Faculty of Applied Bioscience, Tokyo University of Agriculture, 1-1-1 Sakuragaoka, Setagaya-ku, Tokyo, 156-8502 Japan; Advantec.Co., Ltd, 2-7-1 Nishisinjuku, Sinjuku-ku, Tokyo, 163-0703 Japan; Faculty of Applied Bioscience, Nodai Culture Collection Center, Tokyo University of Agriculture, 1-1-1 Sakuragaoka, Setagaya-ku, Tokyo, 156-8502 Japan; Nodai Research Institute, Tokyo University of Agriculture, 1-1-1 Sakuragaoka, Setagaya-ku, Tokyo, 156-8502 Japan

## Abstract

Proanthocyanidins are abundant in peanut skin, and in this study, the antibacterial effects of a peanut skin extract (PSE) against food-borne bacteria were investigated to find its minimum inhibitory concentration. Food-borne gram-positive bacteria, and in particular *Bacillus cereus*, was more sensitive to PSE. In particular, the inhibitory activity of epicatechin-(4β → 6)-epicatechin-(2β → O→7, 4β → 8)-catechin (EEC), a proanthocyanidin trimer from peanut skin, against *B. cereus* was stronger than that of procyanidin A1, a proanthocyanidin dimer. DNA microarray analysis of *B. cereus* treated with EEC was carried out, with a finding that 597 genes were significantly up-regulated. Analysis of the up-regulated genes suggested that EEC disrupted the normal condition of the cell membrane and wall of *B. cereus* and alter its usual nutritional metabolism. Moreover, treatment of *B. cereus* with EEC inhibited glucose uptake, suggesting that EEC affects the cell-surface adsorption.

**Electronic supplementary material:**

The online version of this article (doi:10.1007/s00284-016-1032-x) contains supplementary material, which is available to authorized users.

## Introduction

*Bacillus cereus* is a gram-positive food-borne bacterium, and is widely distributed in the environment, mainly in soil. Thus, foods such as carrot, zucchini, rice, eggs, and milk are a potential risk for *B. cereus* contamination. *B. cereus* are able to withstand low pH conditions such as foods acidified during food processing and conservation. Moreover, *B. cereus* spores are resistant to gastric acidity [4]. Therefore chemical and physical treatments including hydrogen peroxide, NaClO, ozone, and UV light have been used to inactivate *B. cereus* [12].

Recently, it was demonstrated that plant metaboites like polyphenols possess antimicrobial activities [9]. Tea catechins are well-known plant polyphenols and have antimicrobial activities against *B. cereus* [9, 10]. Friedman et al. [9] reported that epigallocatechin gallate, epicatechin-3-gallate, and theaflavin gallate show antimicrobial activities at nanomolar levels, while tea catechins without gallate side chains, (±)-catechin and gallic acid, are all inactive. Accordingly, the authors suggested that the antimicrobial effect of catechins is strongly influenced by their structure. Besides tea catechins, phenolic compounds in cinnamon, olive oil, and strawberry grapes have also been shown to have antimicrobial properties [3, 11, 16]. Although the antimicrobial capacity of phenolic compounds has been studied at the structural and stereochemical levels, little information is available about the physiological responses of bacteria upon exposure to polyphenols.

**Fig. 1:**
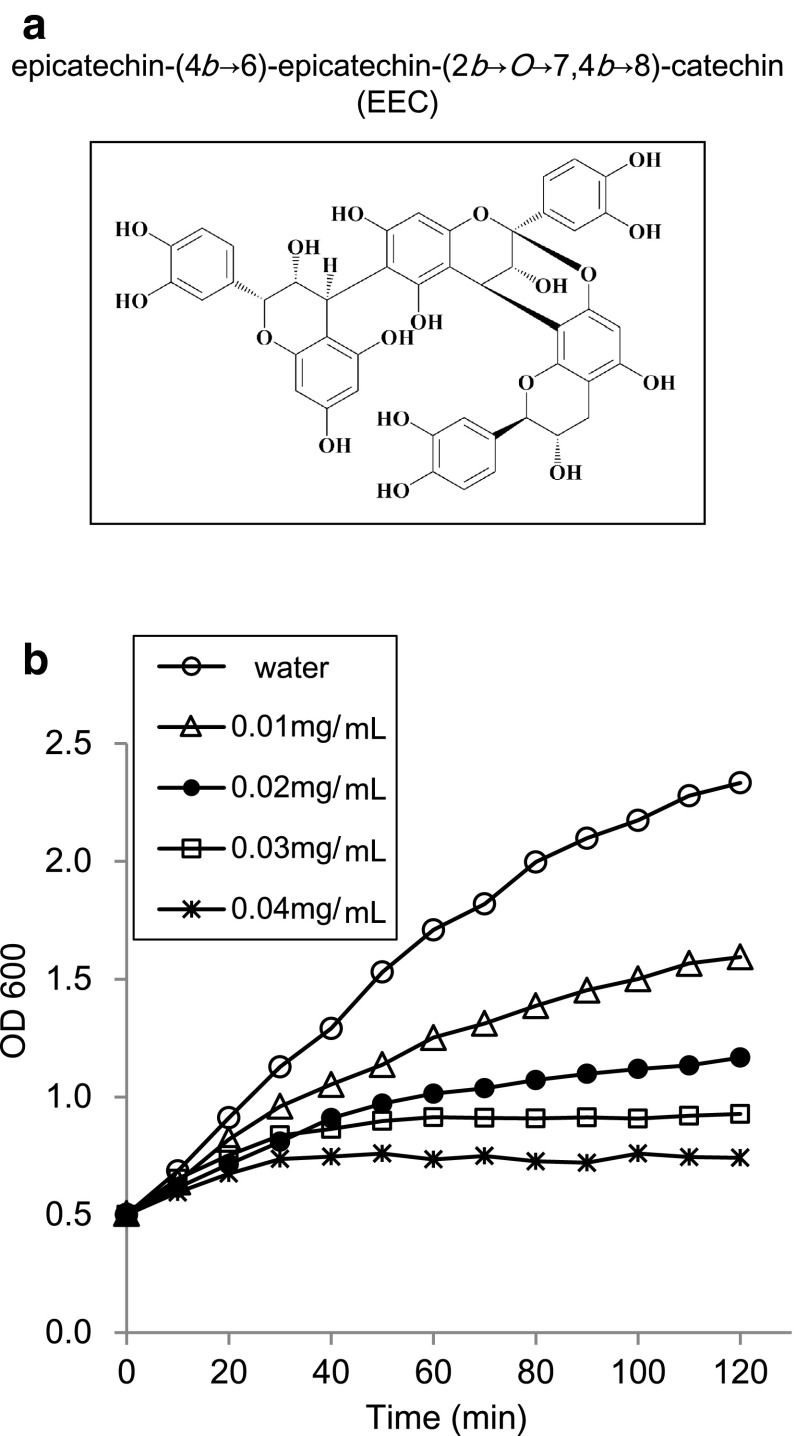
Structure of EEC (**a**) and growth curves of *B. cereus* ATCC 14579 in the presence of different concentrations of EEC (**b**). *B. cereus* was grown in LB medium to an OD_600_ of 0.5 and treated with different concentrations of EEC for 120 min

The red skin of the peanut (*Arachis hypogaea* L.) contains high levels of polyphenols. We have previously demonstrated the antiallergic, hypocholesterolemic, hypoglycemic, and antioxidative effects of peanut skin polyphenols [7, 18, 20, 21]. We also identified procyanidin A1 as a proanthocyanidin dimer and epicatechin-(4β → 6)-epicatechin-(2β → O→7, 4β → 8)-catechin (EEC) as a proanthocyanidin trimer from peanut skin (Fig. 1a). EEC exhibited a more potent cholesterol micelle-degrading activity compared to procyanidin A1, while (+)-catechin had no activity [18]. In addition, the inhibitory effects on sugar digestion enzymes and glucose transport were increased as the degree of polymerization increased [20], and EEC in particular exerted highly hypocholesterolemic and hypoglycemic effects.

Therefore, we assessed the antimicrobial mechanisms of peanut skin polyphenols, particularly EEC. The main goal of this study was to determine the antimicrobial activity of peanut skin polyphenols and to investigate the responses of *B. cereus* in the EEC stress condition using gene expression analysis. In this study, three types of experiments were conducted. First, we compared the activity of peanut skin polyphenols against many bacterial species to clarify their antibacterial spectrum. Next, we performed DNA microarray for genome-wide transcriptional analysis of *B. cereus* ATCC 14,579 in the presence of EEC, and finally, we confirmed the response of *B. cereus* to EEC treatment by quantitative real-time PCR and glucose uptake test.

## Materials and Methods

### Peanut Skin Polyphenol Extraction

Peanut skin extract (PSE) was obtained from peanut skin as previously reported [18]. In brief, peanut skins were boiled in water for 30 min, and precipitate was separated from the supernatant and boiled again in water. This was repeated 5 times, and all supernatants were concentrated *in vacuo* to obtain PSE. Higher- and lower-molecular weight fractions were prepared by ultrafiltration (MWCO 10,000; Advantec, Tokyo). Procyanidin A1 and EEC were separated by chromatography on a TSK-gel Toyopearl HW-40 F column (26 × 800 mm) (Tosoh Bioscience, Tokyo), and YMC-gel ODS-AQ 12S50 column (YMC, Kyoto), as described previously [18, 21]. Total polyphenol content was determined according to the method of Singleton and Rossi [17] with Folin–Ciocalteu’s reagent.

### Microbial Strains

The following strains were used as indicators for the antibacterial testing and were obtained from the NODAI Culture Collection Center (NRIC): *Bacillus* cereus NRIC 0591 (=ATCC14579), *Bacillus subtilis* NRIC 0546 (=168), *Clostridium butyricum* NRIC 0221, *Lactobacillus plantarum* NRIC 1062 (=NBRC 3074), *Micrococcus luteus* NRIC 1094 (=ATCC 4698), *Staphylococcus aureus* NRIC 1135 (=ATCC 11,522), *Streptococcus mutans* NRIC 0528, *Streptococcus sobrinus* NRIC 1694, *Escherichia coli* NRIC 1023 (=NBRC 3301), *Salmonella enterica* subsp. *enterica serovar Typhimurium* NRIC 0820 (=NBRC 13245), *Vibrio fluvialis* NRIC 0818 (=JCM 3752), *Vibrio vulnificus* NRIC 0817 (=JCM 3725), *Vibrio parahaemolyticus* NRIC 0821 (=NBRC 12711), *Yersinia enterocolitica* subsp. *enterocolitica* NRIC 0819 (=JCM 7577).

### Antibacterial Activity Test

Twenty milliliters of the test cultivation medium (Online Resource 1) was applied to a petri dish (90 mm in diameter). After preculture of bacteria in each medium (Online Resource 1), 0.3 mL of the bacterial suspension was added to 4 mL of test cultivation medium before agar has solidified prior to layering on the test cultivation medium. Next, a cylinder (6 × 8 mm) was placed on the dish and different concentrations of 0.2 mL of PSE, procyanidin A1 or EEC were poured into the cylinder. The antibacterial activity test was carried out in test cultivation medium (Nissui, Tokyo, Japan) at 25 or 37 °C (Online Resource 1) for one day, or for two days for *Streptococcus mutans*, *Streptococcus sobrinus*, and *Yersinia enterocolitica* subsp. *Enterocolitica,* or 10 days for *Clostridium butyricum*. The minimum inhibitory concentration (MIC) and the value for each polyphenol or extract against each bacterium was determined from three independent experiments.

### Growth Curves of *B. Cereus* in the Presence of EEC

In liquid culture experiments, 20 mL of LB medium was inoculated with 0.1 % (v/v) of overnight culture. When the OD of the bacterial culture at 600 nm reached 0.5, procyanidin A1 or EEC was added to the culture. After the addition of procyanidin A1 or EEC, the OD_600_ was measured at 10 min intervals.

### Microarray Design

The microarray used in this study was custom made for *B. cereus* using standard Agilent Technologies protocols (https://earray.chem.agilent.com/earray/). The *B. cereus* microarray design was based on the 5234 predicted open reading frames available at NCBI (Accession No. AE016877). Three non-overlapping probes were designed per gene and a final 15242 probes were spotted per array.

### Total RNA Isolation

When the *B. cereus* culture reached an OD_600_ of 0.5, samples for RNA isolation were taken before addition of 0.02 mg/mL EEC and after 30 min of exposure to EEC. Water was added in place of EEC as a control. RNA isolation, DNA removal with DNase I, and RNA purification were done using a Qiagen RNeasy kit according to the manufacturer’s instructions (Qiagen, Tokyo). RNA quality and quantity were determined as described previously [19].

### cDNA Labeling and Microarray Hybridization

The cDNA labeling and microarray hybridization were performed using FairPlay Microarray Labeling kit and Gene Expression Hybridization Kit (Agilent Technologies, Palo Alto, CA). The *B. cereus* microarrays were hybridized (http://www.ncbi.nlm.nih.gov/geo/query/acc.cgi?acc=GSE68767), and scanned after washing in an Agilent microarray scanner (G2565CA).

### Analysis of Microarray Data

Analysis of the scanned images was performed as described previously. In brief, data were normalized to 75th percentile and the difference was found to be significant by unpaired *T*-test (*P* = 0.05). Changes in expression greater than 2-fold, for up-regulated genes, and greater than 0.5-fold, for down-regulated genes were regarded as biologically significant. The genes were then divided into classes based on the Clusters of orthologous groups of proteins (COG) classification.

### Quantitative Real-Time PCR Analysis

One microgram of total RNA (3 independent samples/treatment) was used as a template for first-strand cDNA synthesis using a First-Strand cDNA Synthesis Kit (QuantiTect Reverse Transcription Kit). Quantitative real-time PCR was performed as described previously [19] using the following primer sets: *GTP pyrophosphokinase*, 5′- TCGCTAACCCAAAACGAAAC -3′ and 5′- TGCCCAAAAGTCCATTGC -3′, GenBank Accession No. BC_4341; *lia operon protein* LiaI *homologous gene*, 5′- CGGAGCAGGAGTTGTATATTGG -3′ and 5′- GCTGGCGAATGAGAAAGTG -3′, BC_1435; *Phage shock protein A*, 5′- TGGCACATGCAAATCGTC -3′ and 5′- CACGCTCATGCTCTTCATTC -3′, BC_1436; *Penicillin*-*binding proteins* (*PBP*) 1A, 5′- CGTTAGATCCGAAAGCACAG -3′ and 5′- ATTTTCTCCACGGCCACTAC -3′, BC_2281; *16S ribosomal RNA*, 5′- TGGGGAGCAAACAGGATTAG -3′ and 5′- CCTTTGAGTTTCAGCCTTGC -3′, BC_0007. The amount of gene expression was normalized with the level of 16S ribosomal RNA expression. The relative amounts of gene expression were calculated using the standard curve method, and expressed relative to the control. Differences among all conditions were detected by Tukey’s multiple-range test following a one-way analysis of variance (ANOVA). Statistical analyses were performed with SPSS software.

### Glucose Uptake Assays

Cells were grown in LB medium until the OD_600_ was 0.5, and EEC at a final concentration of 0.05 mg/mL was added. Water was added for control experiments. Measurement of d-glucose uptake was started by the addition of 1 mM d-glucose containing 6.17KBq/mL of [^14^C]-d-glucose (Perkin Elmer, Inc., MA, USA). After incubation for 10, 20, and 30 min at 37 °C, 300 μL aliquots were collected and the reaction was stopped by the addition of 750 μL of ice-cold LB medium containing 140 mM glucose. The cells were collected by filtration through a 0.45-μm pore size membrane filter (Millipore, Ireland), and the filters were then washed with 1 mL of 50 mM ice-cold phosphate buffer (pH6.5). The filters were air-dried, and cell-associated radioactivity was measured in a scintillation counter. All assays were performed in triplicate. Statistical analyses were performed with SPSS software, and between-group differences were detected by T-test.

## Results

### Antibacterial Spectrum of PSE

Peanut skin presented a total of 126.3 mg polyphenols, and 0.3 mg (+)-catechin, 7.1 mg procyanidin A1, and 2.8 mg EEC per g dry skin.

The susceptibility to PSE was significantly different for Gram-positive and Gram-negative bacteria (Table 1). *Bacillus cereus* and *Clostridium butyricum* (MIC, 0.25 mg/mL) showed comparatively strong susceptibility. The MICs of PSE for *Lactobacillus plantarum*, *Micrococcus luteus*, *Staphylococcus aureus* are 7.84, 0.31, 1.16 mg/mL, respectively, when chemically defined protein-free medium was used instead of sensitivity disk agar medium (data not shown). On the other hand, Gram-negative bacteria were not affected by PSE up to a concentration of 5.05 mg/mL.

**Table 1:** Minimum inhibitory concentration (MIC) of PSE

Group	Strain	MIC Polyphenol (mg/mL)
Gram-positive bacteria	*Bacillus cereus* ATCC 14579	0.25
	*Bacillus subtilis* 168	2.58
	*Clostridium butyricum* NRIC 0221	0.25
	*Lactobacillus plantarum* IFO 3074	>5.05
	*Micrococcus luteus* ATCC 4698	>5.05
	*Staphylococcus aureus* ATCC 11522	>5.05
	*Streptococcus mutans* NRIC 0528	>5.05
Gram-negative bacteria	*Escherichia coli* IFO 3301	>5.05
	*Salmonella enterica* subsp. *Enterica serovar Typhimurium* IFO 12529	>5.05
	*Vibrio vulnificus* JCM 3725	>5.05
	*Vibrio parahaemolyticus* NBRC 12711	>5.05
	*Yersinia enterocolitica* subsp. *enterocolitica* JCM 7577	>5.05

### Antibacterial Activity of EEC

To investigate the overall changes in gene expression of *B. cereus* in response to EEC, cells should be treated with sublethal but growth-inhibitory concentrations. As reflected in the growth curves (Fig. 1b), 0.01 mg/mL of EEC inhibited the growth rate about two-fold. In the presence of 0.03 mg/mL EEC, the growth of *B. cereus* was completely inhibited. The effect of EEC concentration on *B. cereus* growth could be seen after 30 min, and was prominent after 60 min. Procyanidin A1, a proanthocyanidin dimer (Online Resource 2), also possessed inhibitory activity towards *B. cereus* growth, but the antibacterial activity was 10-fold less than that of EEC (Online Resource 2). Therefore, we carried out DNA microarray analysis on *B. cereus* after 30 min exposure to 0.02 mg/mL EEC.

### Numbers of Differentially Expressed Genes in Response to EEC

**Fig. 2:**
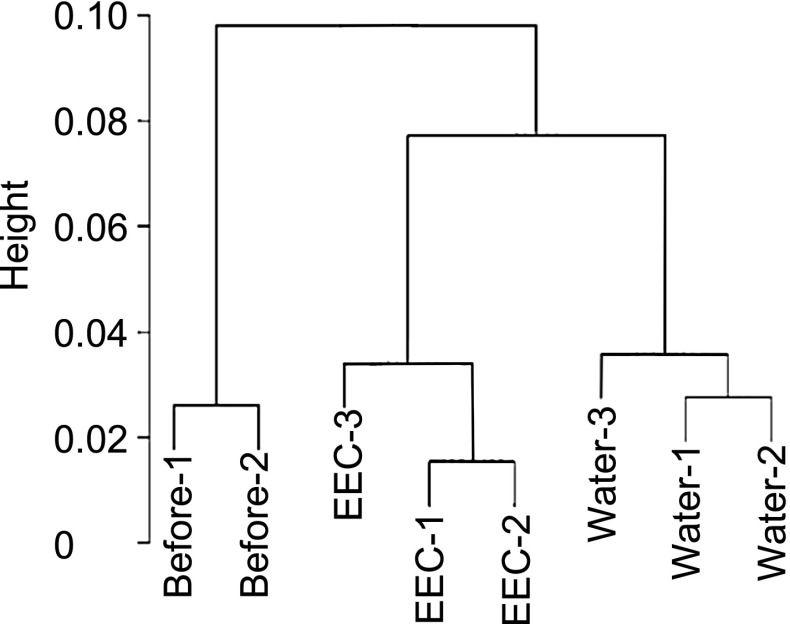
Cluster dendrogram of genes expressed in *B. cereus*. Eight samples from three conditions were used to construct the dendrogram using the ‘pvclust’ function. Before, 0 min after the addition of EEC (OD_600_ = 0.5) to the medium; EEC, 30 min after the addition of 0.02 mg/mL of EEC; Water, 30 min after the addition of water (control)

Three conditions 8 samples were normalized to 75th percentile and constructed a cluster dendrogram by pvclust function in statistical language R, version 2.14.0 (Fig. 2), with the result that the water treatment (control) and the EEC treatment belong to different clusters. Statistical analysis indicated that changes in the expression of 1055 genes, represented by 2181 probes, were significant. Among these genes, 596 were up-regulated and 495 were down-regulated. All the extracted genes by the transcriptomic analysis are shown in Online Resource 3.

### COG Classification

**Fig. 3:**
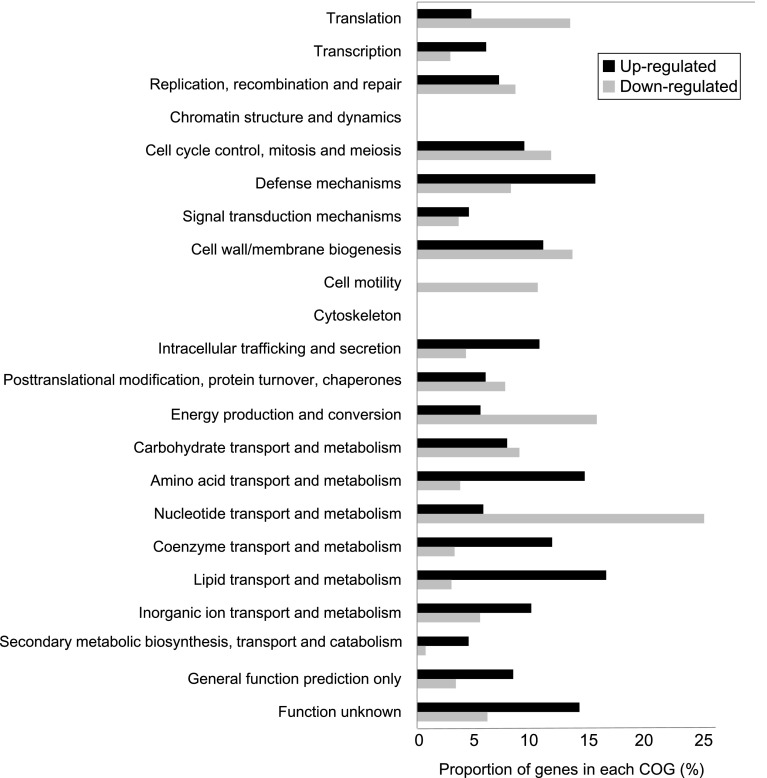
Differentially expressed genes (*P* < 0.05) in the presence of EEC on the basis of COG classification. Some genes belong to more than one category

Figure 3 shows up- and down-regulated genes divided into classes based on the COG classification. Genes up-regulated by EEC treatment were enriched in genes involved with “lipid transport and metabolism” and “defense metabolism”. Genes involved in “amino acid transport and metabolism”, “coenzyme transport and metabolism”, and “lipid transport and metabolism” were also up-regulated. Genes down-regulated by EEC treatment were enriched in genes involved in “nucleotide transport and metabolism”. Genes related to “cell motility” and “energy production and conversion” were also down-regulated.

### Gene Expression Changes of *B. Cereus* ATCC14579 in Response to EEC

**Fig. 4:**
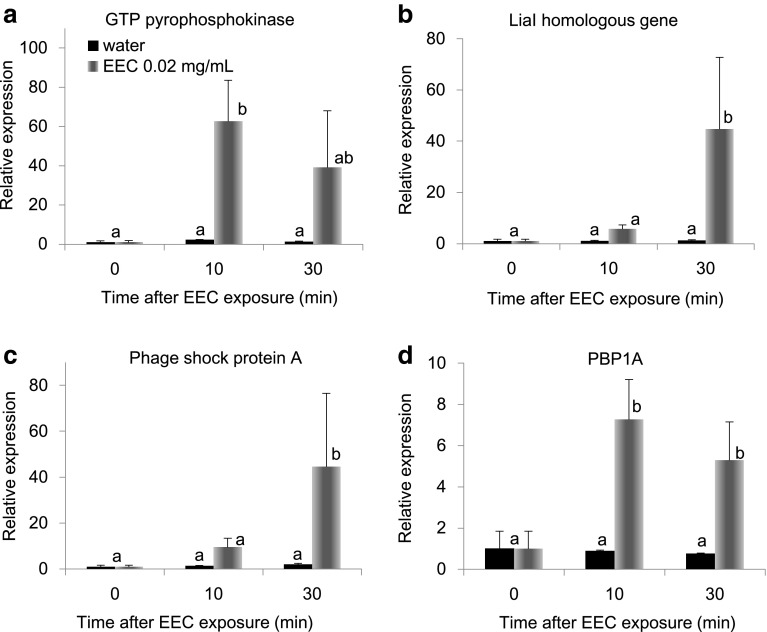
Time-course changes in gene expression in *B. cereus* after EEC exposure. The relative amounts of GTP pyrophosphokinase (**a**), LiaI homologous gene (**b**), phage shock protein A (**c**), and PBP1A (**d**) were normalized with the level of 16S ribosomal RNA expression, and were calculated relative to expression at 0 min.* Error bars* indicate standard deviation (*n* = 3).* Different letters* are significantly different at (*p* < 0.05) according to Tukey’s multiple-range test among all conditions

Table 2 shows the top 50 genes up-regulated by EEC exposure. The most abundant functional category is ABC transporters and include two genes whose expression changed over >6100 fold and 11 more genes that increased >11 fold. The genes encoding GTP pyrophosphokinase that is related to stringent response, was also up-regulated. When analyzed by real-time PCR, the expression level of *GTP pyrophosphokinase* was increased 62.5-fold within 10 min by EEC (Fig. 1a) exposure, and maintained thereafter. (Figure 4a). Genes belonging to the two-component regulatory system, *Lia I homologs* and *phage shock protein A*, were also up-regulated by EEC treatment. Their expression increased 5.7- and 9.5-fold, respectively, 10 min after EEC exposure, and further increased after 30 min of exposure, to 44.7- and 44.5-fold, respectively (Fig. 4b, c). Of the genes related to further polymerization of glycin, *PBP1A* was also prominently increased, and real-time PCR showed a 7.3-fold increase within 10 min and a 5.3-fold increase after 30 min of exposure to EEC (Fig. 4d). The expression changes of three genes encoding bacillibactin synthesis enzymes, 2,3-dihydroxybenzoate-AMP ligase, isochorismatase, and glycine-AMP ligase are also measured and were significantly increased after 10 min of EEC exposure, and then reduced 30 min after treatment (Online Resource 4).

**Table 2:** Top 50 genes up-regulated by EEC exposure

Locus tag	Fold Change	Description	Genes related to
BC_5014	6557.9	Hypothetical exported repetitive protein	ABC transporter
BC_5015	6120.1	Hypothetical exported repetitive protein	ABC transporter
BC_0575	1470.2	Hypothetical protein	
BC_0574	519.0	Hypothetical Membrane spanning protein	ABC transporter
BC_2620	140.5	Penicillin-binding protein transpeptidase	Polymerization of glycan
BC_2985	70.4	Vancomycin B-type resistance protein vanW	Polymerization of glycan
BC_0572	59.3	Two-component response regulator	Two-component regulatory system
BC_0573	56.7	Two-component system histidine kinase	Two-component regulatory system
BC_2621	54.9	Signal peptidase I	
BC_1161	50.4	Foldase protein prsA 2	
BC_2895	50.2	Hypothetical protein	
BC_1760	43.6	3-Oxoacyl-(acyl carrier protein) synthase III	
BC_0754	33.5	Potassium-transporting ATPase B chain	Two-component regulatory system
BC_0755	26.9	Potassium-transporting ATPase C chain	Two-component regulatory system
BC_0785	23.8	Hypothetical protein	
BC_4341	23.1	GTP pyrophosphokinase	Stringent response
BC_2984	21.2	Immune inhibitor A precursor	
BC_1419	20.2	Diaminopimelate decarboxylase	
BC_4775	20.0	Phosphoglycerol transferase	
BC_4251	17.5	Bifunctional homocysteine S-methyltransferase/5,10-methylenetetrahydrofolate reductase protein	
BC_3410	17.4	D-Threo-aldose 1-dehydrogenase	
BC_2603	17.3	Putative uncharacterized protein	
BC_1461	16.5	DNA integration/recombination/invertion protein	
BC_1779	16.3	Ketol-acid reductoisomerase	
BC_2496	15.9	D-Alanyl-d-alanine carboxypeptidase	Polymerization of glycan
BC_0753	15.6	Potassium-transporting ATPase subunit A	Two-component regulatory system
BC_1394	15.3	UPF0180 protein BC_1394	
BC_2169	15.2	Aspartyl-tRNA synthetase	
BC_1777	14.9	Acetolactate synthase large subunit	
BC_1825	14.4	Transposase	
BC_1435	14.4	Hypothetical protein	Two-component regulatory system
BC_4254	14.3	Cystathionine beta-lyase	
BC_3600	14.3	Protease HhoA	
BC_2323	14.1	ABC transporter ATP-binding protein	ABC transporter
BC_3199	14.1	Hypothetical Cytosolic Protein	ABC transporter
BC_1781	14.0	Threonine dehydratase	
BC_4667	13.8	Ankyrin	
BC_4742	13.8	ABC transporter permease protein	ABC transporter
BC_0347	13.5	ABC transporter permease protein	ABC transporter
BC_4830	13.4	ABC transporter permease protein	ABC transporter
BC_4831	13.2	ABC transporter ATP-binding protein	ABC transporter
BC_2977	12.6	Pyrroline-5-carboxylate reductase	
BC_1995	12.4	ABC transporter permease protein	ABC transporter
BC_4545	12.3	Ferrichrome transport system permease protein fhuB	ABC transporter
BC_4743	12.3	ABC transporter ATP-binding protein	ABC transporter
BC_4038	12.0	Methylthioribulose-1-phosphate dehydratase	
BC_0816	11.9	Periplasmic component of efflux system	
BC_4134	11.8	pyrroline-5-carboxylate reductase	
BC_0814	11.7	ABC transporter permease protein	ABC transporter
BC_2272	11.5	Peptidylprolyl isomerase

### Inhibitory Effect of EEC on Glucose Uptake

**Fig. 5:**
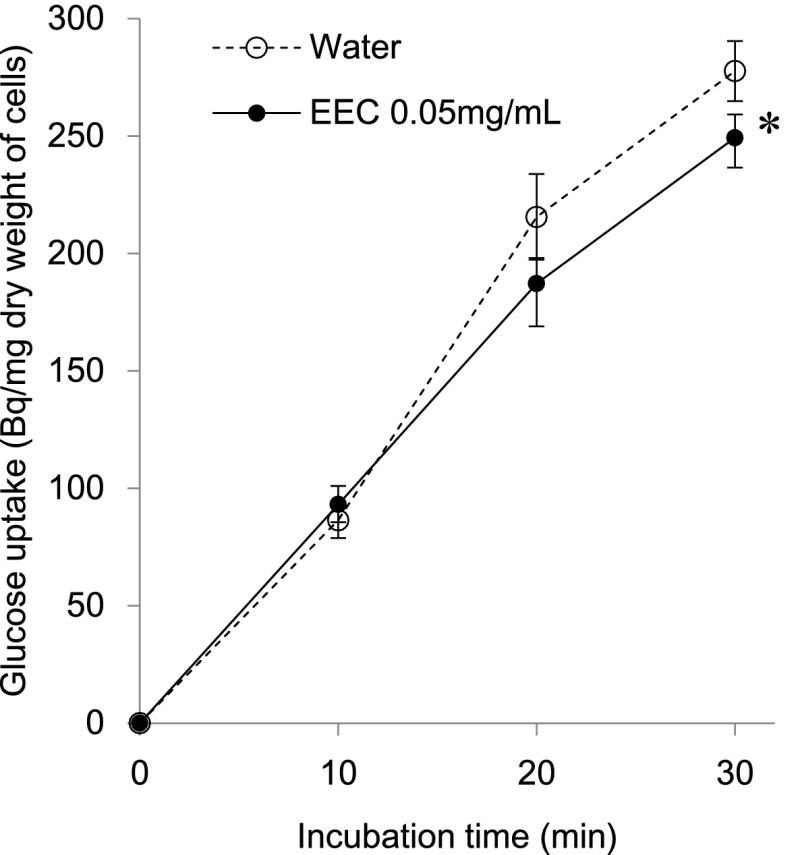
Effect of EEC on glucose uptake by *B. cereus. B. cereus* was grown in LB medium to an OD_600_ of 0.5, and 0.05 mg/mL of EEC, and 1 mM d-glucose containing 6.17KBq/mL of [^14^C]-d-glucose were added to the medium. After incubation for 10, 20, and 30 min at 37 °C, the reaction was stopped, and cells were collected by filtration. The filters were air-dried, and cell-associated radioactivity was measured in a scintillation counter.* Error bars* indicate standard deviation (*n* = 3).* Asterisk* indicates significant difference at *P* < 0.05

To further examine the effect of EEC on nutritional metabolism, we studied glucose uptake. Figure 5 shows the level of glucose uptake in *B. cereus* after EEC exposure. The glucose uptake level was significantly decreased after 30 min incubation with 0.05 mg/mL of EEC, although no difference was observed after 10 min incubation. There was no difference in glucose uptake between the control and EEC when used at 0.02 mg/mL EEC, the concentration used for DNA microarray analysis (data not shown).

## Discussion

The antibacterial spectrum of PSE showed that the inhibitory effect of PSE was more effective against Gram-positive than Gram-negative bacteria (Table 1). We hypothesize that the PSE may more easily access the cell membrane and the cell wall of gram-positive bacteria. More than 5.05 mg/mL PSE might inhibit the growth of gram-negative bacteria including *E. coli*, but results were not obtained, because osmotic pressure had been produced between solid medium and PSE solution, and hence water filled the inside of the cylinder. On the other hand, less than 5.05 mg/mL PSE were enough to inhibit growth of *Lactobacillus plantarum*, *Micrococcus luteus*, and *Staphylococcus aureus* when chemically defined protein-free medium was used. We expected that components of medium could affect the sensitivity to PSE in Gram-positive bacteria. EEC inhibited the growth of *B. cereus* to a greater extent than procyanidin A1 (Online Resource 2). Although Verstraeten et al. [22] reported that proanthocyanidin dimers and trimers from cacao and peanut skin can interact with membrane phospholipids and decrease membrane fluidity to a similar degree, we suggest EEC, the proantocyanidin trimer, exerts stronger antibacterial activity than procyanidin A1, a proantocyanidin dimer.

Exposure of *B. cereus* to EEC altered the expression of genes related to stringent response, which aids in survival during nutrient limitation. Bacteria respond to nutritional starvation by producing guanosine 5′-diphosphate (or 5′-triphosphate) 3′-diphosphate (ppGpp) in the cell [14, 15]. This compound regulates the activities of various enzymes that mediate nucleotide metabolism, transcription, translation, and DNA replication [24]. Synthesis of ppGpp is mediated by GTP pyrophosphokinase using ATP and GDP(GTP) and its hydrolysis to GDP(GTP) and pyrophosphate. In this study, the gene encoding GTP pyrophosphokinase was induced by EEC exposure (Fig. 4a), suggesting that ppGpp would regulate amino acid, lipid, coenzyme, and nucleotide metabolism and translation (Fig. 3).

The gram-positive cell envelope consists of two functional layers (compared with three in gram-negative bacteria): a cytoplasmic membrane surrounded by a thick cell wall [2]. EEC also affected the expression of the two-component signal transduction system (TCS). Bacteria possess the LiaSR system of the TCS, which responds to cell wall antibiotics, and interferes with perturbation of the cytoplasmic membrane [13]. The primary target gene of LiaSR is *liaIH*, and its physiological role is largely unknown, though Lia H is a member of the phage shock protein family [13]. Recently, several mechanisms were proposed to explain the antimicrobial activity of the A-type proanthocyanidin, such as the destabilization of the cytoplasmic membrane and the permeabilization of the cell membrane [5, 6]. Bernal et al. [1] provided evidence that epicatechin gallate binds to the cytoplasmic membrane of methicillin-resistant *Staphylococcus aureus* and penetrates deep into the hydrophobic region of the bilayer. As a result, ECg reduces membrane fluidity and disrupts cell wall synthesis. In this study, expression of *LiaI homologous genes* and *phage shock protein* were slightly increased after 10 min of EEC exposure, but significantly increased after 30 min (Fig. 4b, c). Genes related to polymerization of glycan were also increased by EEC exposure. The polymerization and cross-linking of peptidoglycan is mediated by PBPs. RT-PCR analysis revealed that *PBP1A*, was increased 10 min after EEC administration (Fig. 4d). We suggest that EEC may disrupt the normal condition of the cell membrane and the cell wall of *B. cereus.* It can be presumed that the destabilization of the cytoplasmic membrane would affect the membrane-associated enzymes and certain intracellular transport mechanisms. Thus, we tested the influence of EEC on glucose uptake by *B cereus*, and predictably, EEC blocked the glucose transporter or affected the transmembrane condition, and reduced the level of glucose uptake (Fig. 5).

It is known that one of the mechanisms of bacterial growth inhibition is deprivation of essential mineral micronutrients, such as iron and zinc, via proanthocyanidin chelation with metals [5, 6]. Many bacteria secrete siderophores [23], low molecular weight carriers with high affinity for Fe^3+^ to grow in iron-deficient substrates. Although Engels et al. [8] demonstrated that the inhibitory activities of gallotannins are attributable to their strong affinity for iron, and likely relate to the inactivation of membrane-bound proteins, our results suggest that iron deprivation would not be directly responsible to the antimicrobial activity of EEC against *B. cereus.*

Collectively, our results suggest that the peanut skin is a potential source of antibacterial agents with bacteriostatic activity against *B. cereus*. EEC could be keeping *B. cereus* at low levels cause the disruption of normal conditions in the cell membrane and the cell wall and affect the function of membrane-binding protein. Additional genetic and biochemical analysis of *B. cereus* about toxin production and spore formation will be needed to elucidate the bacteriostatic activity of EEC. It is also important to analyze the effectiveness of EEC when *B. cereus* is in a food commodity.

## Electronic supplementary material

Below is the link to the electronic supplementary material.

Supplementary material 1 (PPTX 47 kb)

Supplementary material 2 (PPTX 95 kb)

Supplementary material 3 (XLSX 414 kb)

## Abbreviations

COG: Clusters of orthologous groups
EEC: Epicatechin-(4β → 6)-epicatechin-(2β → O→7, 4β → 8)-catechin
MIC: Minimum inhibitory concentration
NRIC: NODAI Culture Collection Center
PBP: Penicillin-binding proteins
PSE: Peanut skin extract
TCS: Two-component signal transduction system
