# Supplementary material for: Bacillus cereus Response to a Proanthocyanidin Trimer, a Transcriptional and Functional Analysis
Source: Curr Microbiol. 2016 Apr 9;73:115–23. doi: 10.1007/s00284-016-1032-x (PMC4899491; doi:10.1007/s00284-016-1032-x)
Supplement: Supplementary file 1 — Supplementary material 1 (PPTX 47 kb) [file 284_2016_1032_MOESM1_ESM.pptx]

## Slide 1
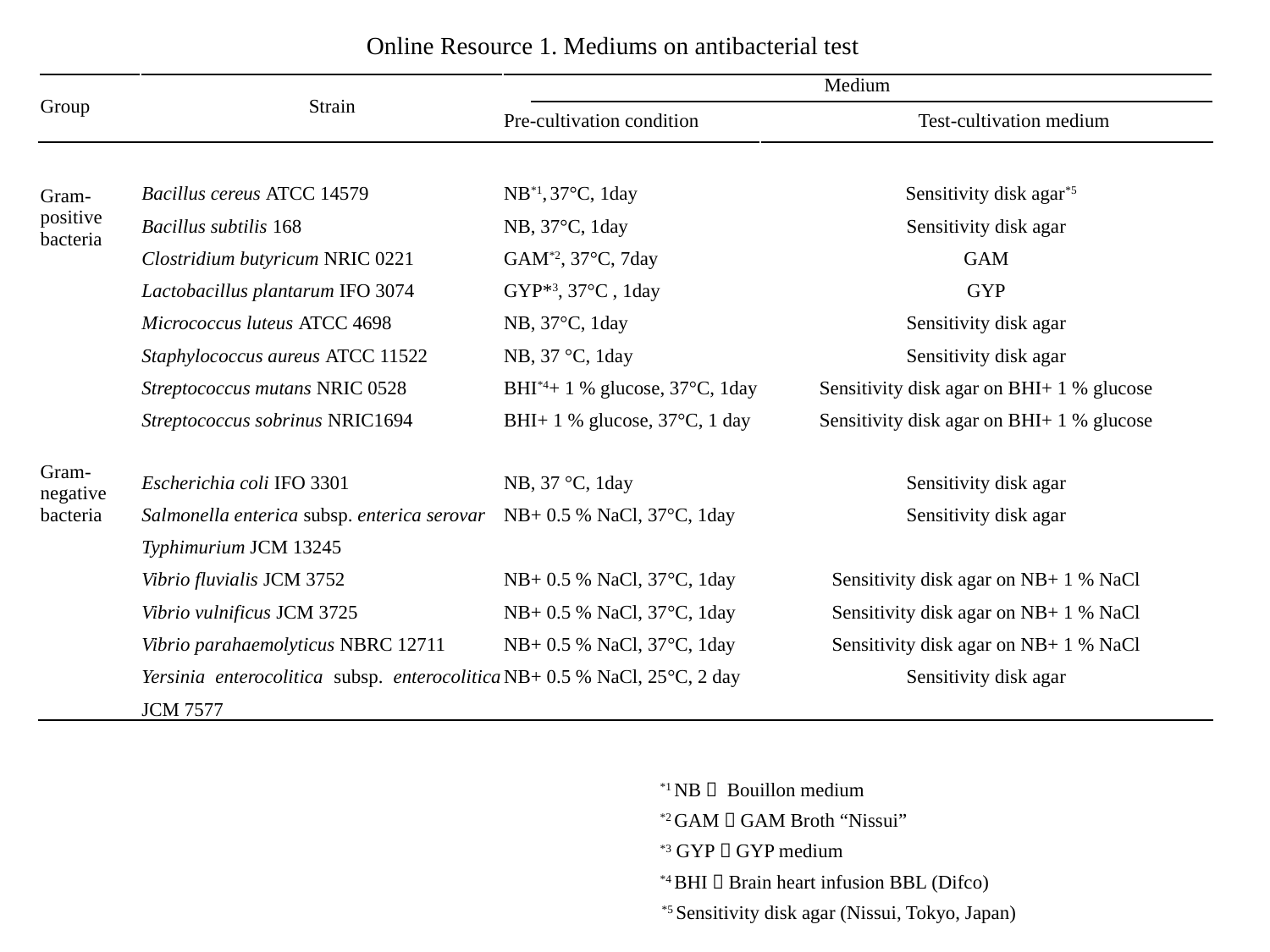

Online Resource 1. Mediums on antibacterial test
| Group | Strain | Medium | | |
| --- | --- | --- | --- | --- |
| | | Pre-cultivation condition | | Test-cultivation medium |
| Gram- positive bacteria | Bacillus cereus ATCC 14579 Bacillus subtilis 168 Clostridium butyricum NRIC 0221 Lactobacillus plantarum IFO 3074 Micrococcus luteus ATCC 4698 Staphylococcus aureus ATCC 11522 Streptococcus mutans NRIC 0528 Streptococcus sobrinus NRIC1694 | NB\*1, 37°C, 1day NB, 37°C, 1day GAM\*2, 37°C, 7day GYP\*3, 37°C , 1day NB, 37°C, 1day NB, 37 °C, 1day BHI\*4+ 1 % glucose, 37°C, 1day BHI+ 1 % glucose, 37°C, 1 day | Sensitivity disk agar\*5 Sensitivity disk agar GAM GYP Sensitivity disk agar Sensitivity disk agar Sensitivity disk agar on BHI+ 1 % glucose Sensitivity disk agar on BHI+ 1 % glucose | |
| Gram- negative bacteria | Escherichia coli IFO 3301 Salmonella enterica subsp. enterica serovar Typhimurium JCM 13245 Vibrio fluvialis JCM 3752 Vibrio vulnificus JCM 3725 Vibrio parahaemolyticus NBRC 12711 Yersinia enterocolitica subsp. enterocolitica JCM 7577 | NB, 37 °C, 1day NB+ 0.5 % NaCl, 37°C, 1day  NB+ 0.5 % NaCl, 37°C, 1day NB+ 0.5 % NaCl, 37°C, 1day NB+ 0.5 % NaCl, 37°C, 1day NB+ 0.5 % NaCl, 25°C, 2 day | Sensitivity disk agar Sensitivity disk agar   Sensitivity disk agar on NB+ 1 % NaCl Sensitivity disk agar on NB+ 1 % NaCl Sensitivity disk agar on NB+ 1 % NaCl Sensitivity disk agar | |
*1 NB： Bouillon medium
*2 GAM：GAM Broth “Nissui”
*3 GYP：GYP medium
*4 BHI：Brain heart infusion BBL (Difco)
*5 Sensitivity disk agar (Nissui, Tokyo, Japan)
