# Supplementary material for: Bacillus cereus Response to a Proanthocyanidin Trimer, a Transcriptional and Functional Analysis
Source: Curr Microbiol. 2016 Apr 9;73:115–23. doi: 10.1007/s00284-016-1032-x (PMC4899491; doi:10.1007/s00284-016-1032-x)
Supplement: Supplementary file 2 — Supplementary material 2 (PPTX 95 kb) [file 284_2016_1032_MOESM2_ESM.pptx]

## Slide 1
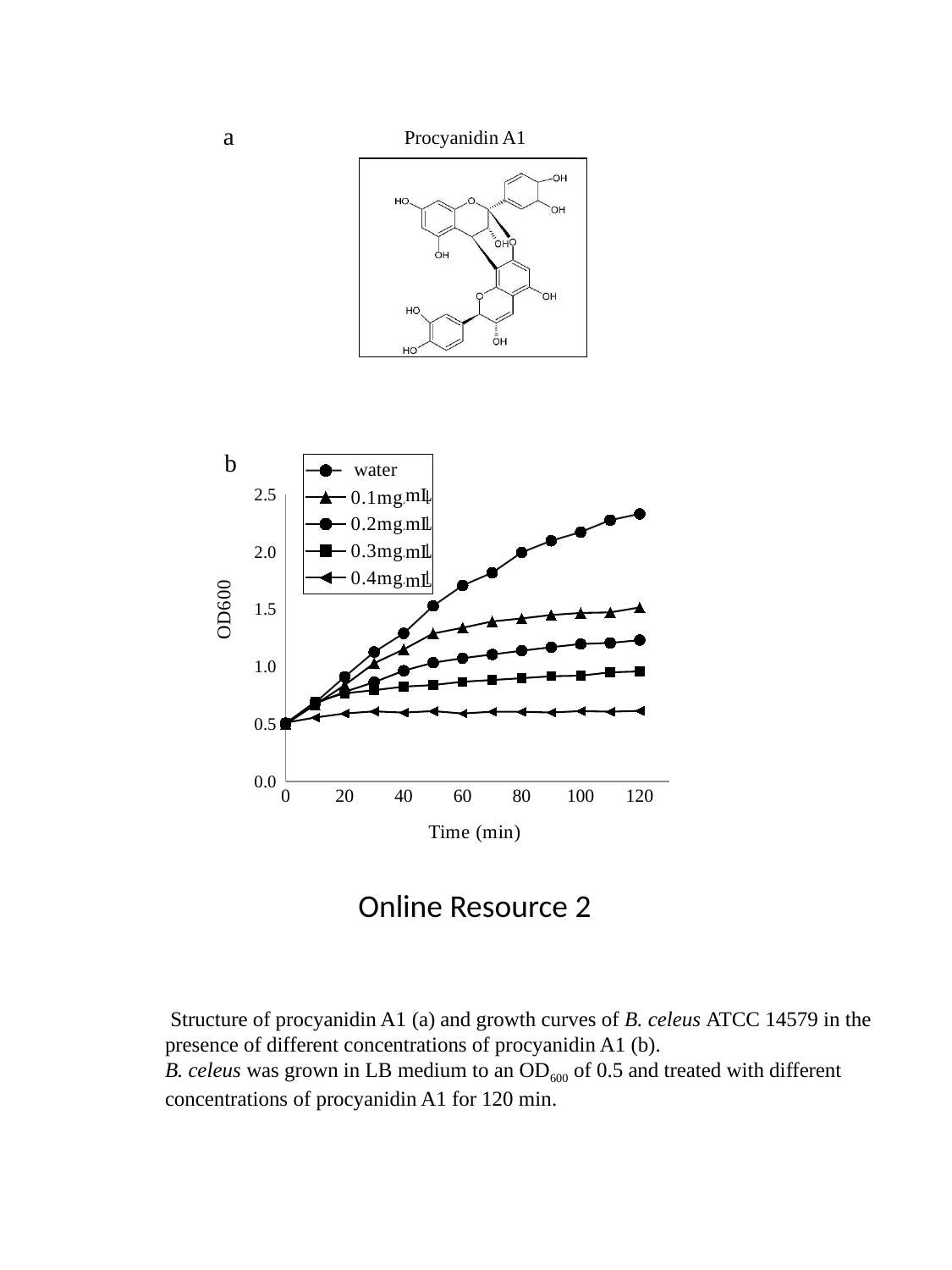

a
Procyanidin A1
### Chart
| Category | control | 0.1mg/ml | 0.2mg/ml | 0.3mg/ml | 0.4mg/ml |
|---|---|---|---|---|---|water
b
mL
mL
mL
mL
Online Resource 2
 Structure of procyanidin A1 (a) and growth curves of B. celeus ATCC 14579 in the presence of different concentrations of procyanidin A1 (b).
B. celeus was grown in LB medium to an OD600 of 0.5 and treated with different concentrations of procyanidin A1 for 120 min.

## Slide 2
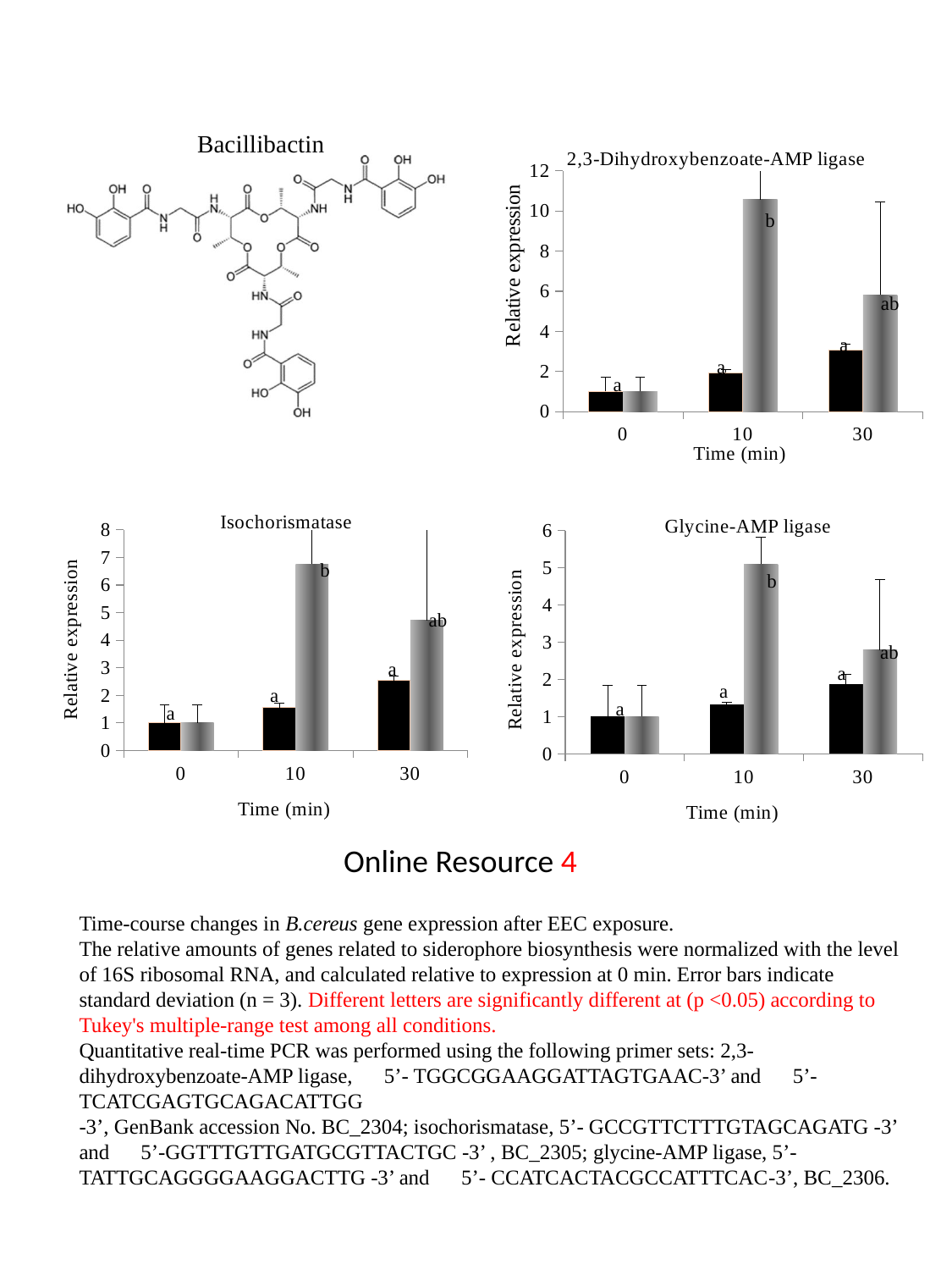

Bacillibactin
### Chart: 2,3-Dihydroxybenzoate-AMP ligase
| Category | | |
|---|---|---|
| 0 | 1.0 | 1.0 |
| 10 | 10.565144473810241 | 1.9315805499878793 |
| 30 | 5.81830918045775 | 3.0853048820145332 |b
ab
a
a
a
### Chart: Isochorismatase
| Category | | |
|---|---|---|
| 0 | 1.0 | 1.0 |
| 10 | 1.5587744722469987 | 6.758318392075637 |
| 30 | 2.531365830781756 | 4.725772657282207 |
### Chart: Glycine-AMP ligase
| Category | | |
|---|---|---|
| 0 | 1.0 | 1.0 |
| 10 | 1.3182645694790256 | 5.0935845761663385 |
| 30 | 1.8680031397886443 | 2.7841631939592397 |b
b
ab
ab
a
a
a
a
a
a
Online Resource 4
Time-course changes in B.cereus gene expression after EEC exposure.
The relative amounts of genes related to siderophore biosynthesis were normalized with the level of 16S ribosomal RNA, and calculated relative to expression at 0 min. Error bars indicate standard deviation (n = 3). Different letters are significantly different at (p <0.05) according to Tukey's multiple-range test among all conditions.
Quantitative real-time PCR was performed using the following primer sets: 2,3-dihydroxybenzoate-AMP ligase,　5’- TGGCGGAAGGATTAGTGAAC-3’ and　5’-TCATCGAGTGCAGACATTGG
-3’, GenBank accession No. BC_2304; isochorismatase, 5’- GCCGTTCTTTGTAGCAGATG -3’ and　5’-GGTTTGTTGATGCGTTACTGC -3’ , BC_2305; glycine-AMP ligase, 5’- TATTGCAGGGGAAGGACTTG -3’ and　5’- CCATCACTACGCCATTTCAC-3’, BC_2306.
